# The Optimal Image Date Selection for Evaluating Cultivated Land Quality Based on Gaofen-1 Images

**DOI:** 10.3390/s19224937

**Published:** 2019-11-13

**Authors:** Ziqing Xia, Yiping Peng, Shanshan Liu, Zhenhua Liu, Guangxing Wang, A-Xing Zhu, Yueming Hu

**Affiliations:** 1 College of Natural Resources and Environment, South China Agricultural University, Guangzhou 510642, Chinapyppyp@stu.scau.edu.cn (Y.P.); ShanshanL@stu.scau.edu.cn (S.L.); gxwang@siu.edu (G.W.); azhu@wisc.edu (A.-X.Z.); 2 Department of Geography and Environmental Resources, Southern Illinois University Carbondale (SIUC), Carbondale, IL 62901, USA; 3 Department of Geography, University of Wisconsin-Madison, Madison, WI 53706, USA; 4 Guangdong Provincial Key Laboratory of Land Use and Consolidation, South China Agricultural University, Guangzhou 510642, China; 5 Guangdong Province Engineering Research Center for Land Information Technology, South China Agricultural University, Guangzhou 510642, China; 6 Key Laboratory of Construction Land Transformation, Ministry of Land and Resources, South China Agricultural University, Guangzhou 510642, China

**Keywords:** cultivated land quality, Gaofen-1, optimal image date

## Abstract

This study proposes a method for determining the optimal image date to improve the evaluation of cultivated land quality (CLQ). Five vegetation indices: leaf area index (LAI), difference vegetation index (DVI), enhanced vegetation index (EVI), normalized difference vegetation index (NDVI), and ratio vegetation index (RVI) are first retrieved using the PROSAIL model and Gaofen-1 (GF-1) images. The indices are then introduced into four regression models at different growth stages for assessing CLQ. The optimal image date of CLQ evaluation is finally determined according to the root mean square error (RMSE). This method is tested and validated in a rice growth area of Southern China based on 115 sample plots and five GF-1 images acquired at the tillering, jointing, booting, heading to flowering, and milk ripe and maturity stage of rice in 2015, respectively. The results show that the RMSEs between the measured and estimated CLQ from four vegetation index-based regression models at the heading to flowering stage are smaller than those at the other growth stages, indicating that the image date corresponding with the heading to flowering stage is optimal for CLQ evaluation. Compared with other vegetation index-based models, the LAI-based logarithm model provides the most accurate estimates of CLQ. The optimal model is also driven using the GF-1 image at the heading to flowering stage to map CLQ of the study area, leading to a relative RMSE of 14.09% at the regional scale. This further implies that the heading to flowering stage is the optimal image time for evaluating CLQ. This study is the first effort to provide an applicable method of selecting the optimal image date to improve the estimation of CLQ and thus advanced the literature in this field.

## 1. Introduction

Cultivated land provides food and is an important resource and material basis for human survival and development [1,2]. The evaluation of cultivated land quality (CLQ) plays an important role in improving and protecting cultivated land and is related to land productive potential [3,4]. Although traditional measurement methods, including field and laboratory measurements, provide an accurate evaluation of CLQ, they are time-consuming and costly [5,6,7], and do not meet the need of modern cultivated land management. Remote sensing technology offers a unique means for rapid evaluation of CLQ at a regional scale with a low cost [4]. However, not all remote sensing images in growth stages of crop can reflect CLQ due to the effects of complex crop types and crop growth environment. Therefore, it is very important to select an optimal image date of remote sensing images for evaluating CLQ.

Current studies on the determination of optimal image dates mainly focus on crop identification, determination of crop harvest date, and estimation of crop yield [8,9,10]. For crop identification, the optimal image dates are often selected by exploring the relationship between spectral vegetation indices and crops at phenology [11,12,13]. For example, Wang et al. (1991) compared the perpendicular vegetation index (PVI) of different crops during the growth stage of cotton and found that the dates of images obtained at the budding stage and the middle stage of boll opening were optimal to identify cotton [14]. Qi et al. (2008) analyzed the phenology and spectral separability of winter wheat at different growth stages and found that early April was the optimal image date for identifying winter wheat [8]. The research of Li et al. (2015) used J-M distances of canopy spectral reflectance at different sowing dates and found that 19 December was the optimal image date for monitoring winter wheat sowing using remote sensing data in the northern plain of North China [15].

Moreover, there have been some studies [16,17] that described methods using spectral indices for selecting the optimal harvest date for crops. Meng et al. (2015) analyzed the relationship between field-observed optimal soybean harvest dates and image-derived spectral indices such as normalized difference vegetation index (NDVI) and normalized difference water index (NDWI) at different times to select the optimal harvest date of soybean and found that the optimal harvest time was 2–3 weeks earlier than the general harvest date [9]. Jin et al. (2019) assessed corn kernel moisture and identified an effective biochemical indicator (i.e., canopy chlorophyll content) of the optimal harvest date using remote sensing, which provided a new avenue for effectively predicting crop optimal harvest date using multispectral imagery across large areas [18].

In addition, substantial studies [19,20,21] have been conducted to select the optimal image dates for crop yield estimation by analyzing the relationship between spectral indices and crop yield. For example, Guindin-Garcia (2010) found that leaf area index (LAI) at the mid-grain filling period was the best predictor of corn grain yield by analyzing corn yield formation, a key crop biophysical parameter, and optimum developmental stages during the growing season [10]. Based on the correlation analysis between NDVI and crop yield at different growth stages, Huang et al. (2014) found that the optimal image dates for estimating the yields of rice, winter wheat, and corn were the heading, flowering, and filling stage, respectively [22].

The previous studies show that there have been no studies conducted for the determination of optimal image dates of CLQ evaluation. It is also very difficult to use the optimal image dates obtained for crop identification, crop harvest, and crop yield estimation to evaluate CLQ because CLQ is a comprehensive measure of cultivated land potential productivity and affected by many factors, and also due to different objects with different spectral responses. Moreover, the previously obtained optimal image dates for crop harvest and yield estimation were determined using the widely used image-derived vegetation indices such as NDVI, EVI, etc., and agronomic parameters such as chlorophyll content. However, the previous studies ignored LAI, the most important canopy structure parameter used in process-based models of crop biogeochemistry. The image-derived vegetation indices were also not compared with the agronomic parameters.

Therefore, the contribution of this work is a method that can be used to determine the optimal image date for CLQ evaluation in a study area. In the study, five spectral vegetation indices including LAI, NDVI, difference vegetation index (DVI); enhanced vegetation index (EVI); and ratio vegetation index (RVI) are retrieved using the PROSAIL model and Gaofen-1 (GF-1) images from 3 August 2015 to 24 October 2015 and corresponding to five growth stages of rice. The integrations of the indices with linear, exponential, logarithmic and power model are compared to estimate CLQ. The optimal image date is determined by analyzing the spectral response relationships between the spectral vegetation indices and CLQ based on 70 training samples from different growth stages of rice. The obtained optimal image date is validated by comparing its CLQ estimates with measured values of 45 validation samples in the study area. The results show that the image date corresponding to the heading to flowering stage is optimal and could be used to evaluate CLQ with RMSE of 183.11 and RRMSE of 14.09%. Therefore, it is expected that the optimal image date can be used to accurately evaluate CLQ.

## 2. Materials and Methods

### 2.1. Study Area

**Figure 1:**
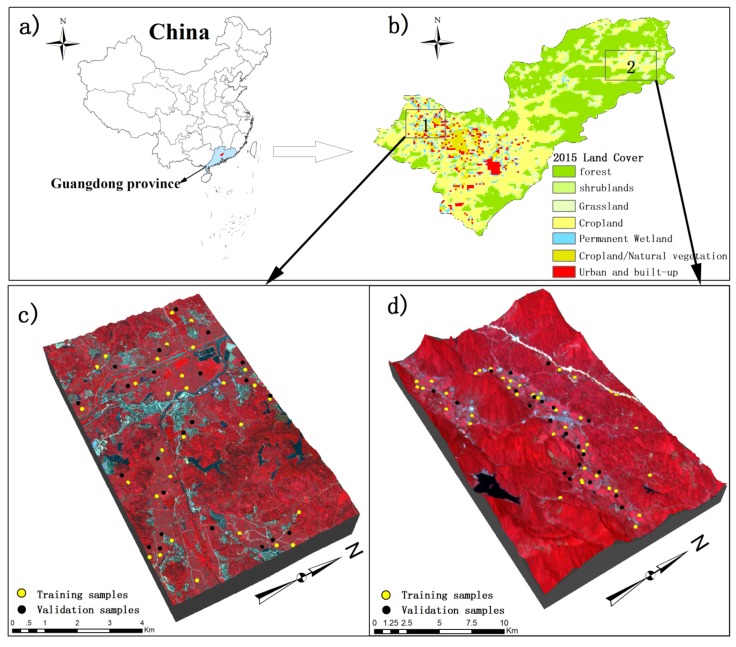
(**a**) Location of the study area in China; (**b**) 500-m spatial resolution MODIS land cover map from a MCD12 product for the study area; (**c**,**d**) the spatial distributions of 115 sample plots within 2 blocks of the study area (70 training sample plots in yellow and 45 validation sample plots in black).

The study area (Figure 1) is located in the Conghua District of Guangzhou, Guangdong Province, China (113°17′–114°04′ E, 23°22′–23°56′ N). The study area has a humid subtropical monsoon climate and is characterized by warm winters, hot summers, little frost and snow, and sufficient rain and sunshine. The annual average temperature is 21.6 °C and the annual precipitation is 2176.3 mm. Rice is one of the major crops in the study area and can be planted in two seasons each year. As a commercially developed area, there is the degradation of cultivated land caused by industrial and agricultural pollution in this area. In this study, cultivated land with rice planted is selected because of economic importance and rice’s contribution in food security in the study area. In this experiment, 70 training sample plots (marked in yellow in Figure 1) and 45 validation sample plots (marked in black in Figure 1) are selected from 2 blocks and located using a global positioning system (GPS) receiver on the GF-1 image.

### 2.2. Sample Data

In this study, a total of 115 sample plots are selected based on a stratified sampling design with soil types and land use classes (Figure 1c,d). Soil samples of about 300 g are collected at a soil layer depth of 0–20 cm. Each of the sample plots have an area of 8 m × 8 m corresponding to the spatial resolution of GF-1 data. Within each of the sample plots, soil samples are collected at five points and put together to meet the requirement for GF-1 images at 8 m spatial resolution. One of the five points is located at the plot center and other four points are allocated along the diagonal lines of the plot and with an equal distance between the points. The 115 samples are processed to obtain CLQ data in the Guangdong Provincial Key Laboratory of Land Use and Consolidation, South China Agricultural University, Guangzhou, China. In this study, the CLQ is the agriculture land utilization quality grade, represents natural conditions and degree of anthropogenic use of cultivated land [23,24,25], and involves the quantification and grading of CLQ indices as follows:

(1) $$Y_{i}=\sum \alpha_{tj}\times \frac{\sum_{1}^{m}w_{k}\times f_{ijk}}{100}\times \beta_{j}\times \frac{Y_{ij}}{Y_{j.max}}$$

where $Y_{i}$ is the *i*th sample utilization quality grade index, $\alpha_{tj}$ is the solar and temperature (climatic) productivity potential index of the *j*th appointment crop, where it is constant (=1337) acquired from the GB/T 28407-2012 in this study area, $w_{k}$ is the kth factor weight, $f_{ijk}$ is the kth factor score at the *i*th sample of the *j*th appointment crop, $\beta_{j}$ is the yield ratio coefficient of the jth appointment crop, $Y_{ij}$ is the unit yield of the *j*th appointment crop at *i*th sample under normal input, $Y_{j.max}$ is the highest unit yield of the *j*th appointment crop in the province. In the study area, 13 factors are considered according to the GB/T 28407-2012, including soil profile configuration, soil texture, soil organic matter content, soil layer thickness, soil pH value, terrain, slope, groundwater level, drainage condition, irrigation assurance rate, surface rock outcrop, barrier layer depth, and salinization degree. Furthermore, the factor score ($f_{ijk}$) is obtained from the GB/T 28407-2012. With reference to the GB/T 28407-2012, the CLQ values are divided into three grades (low: 0–600, medium: 600–1400, and high: 1400–3000) in this study. Out of the 115 soil samples, 70 sample plots are randomly selected and used to determine the optimal image date for CLQ evaluation and the left 45 sample plots are employed to evaluate the accuracy of the CLQ values estimated using the optimal image date.

In the way similar to soil sample collection, the LAI values within 115 sample plots are obtained by LAI-2000 Plant Canopy Analyzers (LI-COR, Inc., Lincoln, NE, USA) in the year and season consistent with the time of the acquired GF-1 image. According to the observation data of rice growth in southern China [26], the growth of rice is divided into the following stages: transplanting to turning green, tilling stage, jointing stage, booting stage, heading to flowering stage, and milk ripe and maturity stage [27]. Of five stages, the transplanting to turning green stage is not considered because the values of image-derived spectral indices are too small to be detected due to the influence of water on spectral reflectance of rice in this study. Thus, a total of five growth stages are taken into account (shown in Table 1). Other parameters used in the PROSAIL model are derived from the Leaf Optical Properties EXperiment 93 (LOPEX93) database [28], including leaf structure parameter, pigment content, water content, and contents of other components.

### 2.3. GaoFen-1 Data and Pre-Processing

GF-1 is the first of a series of high-resolution optical Earth observation satellites of the China National Space Administration and aims at advancing the Earth observation system. GF-1 is equipped with two 2 m Pan/8 m multi-spectral cameras and a four 16 m multi-spectral medium-resolution and wide-field view (WFV) camera set. In this study, five high quality images covering the study region from 3 August 2015 to 24 October 2015 during the five growth stages of rice are acquired (Table 1) from the China Centre for Resources Satellite Data and Application (http://218.247.138.119:7777/DSSPlatform/index.html). The images consist of four multi-spectral bands, including band 1-Blue: 0.45–0.52 µm, band 2-Green: 0.52–0.59 µm, band 3-Red: 0.63–0.69 µm, and band 4–Near Infrared (NIR): 0.77–0.89 µm.

Pre-processing the GF-1 images is performed using the ENVI 5.3 software, including radiometric calibration, atmospheric correction, geometric correction, and resampling. The radiance calibration is to convert the digital number (DN) values of the raw images to surface spectral reflectance values based on the equation L=Gain×DN+Bias, where L is the radiance, and Gain and Bias are the calibration coefficients. The values of radiance calibration parameters are shown in Table 2. The atmospheric corrections of the GF-1 data are conducted using the FLAASH model. This model is built with ENVI input software for atmospheric correction of the multispectral and hyper spectral data in the visible-near infrared and shortwave infrared range (up to 3000 nm) [29,30]. The geometric corrections are made with a total of 45 ground control points measured using GPS and least squares transformation. Then, GF-1 WFV bands with 16 m resolution are resampled using the nearest neighbor method to generate GF-1 data with 8 m resolution. The images are finally geo-referenced to the universal transverse mercator (UTM) projection and coordinate system with an allowed root mean square error (RMSE) less than one pixel.

### 2.4. Methods

#### 2.4.1. Prediction of Leaf Area Index by the PROSAIL Model

In this study, the field measured LAI values are not sufficient for generating the spatial distribution of LAI. Therefore, the PROSAIL model is driven to construct the look-up-table (LUT) of the canopy reflectance and LAI according to the adjustment of the LAI parameter step (Table 3). Then, representative LAI estimates of the pixels are acquired from the LUT and considered as references of ground truth. The PROSAIL model combines the PROSPECT leaf optical property model and SAIL canopy bidirectional reflectance model (http://teledetection.ipgp.jussieu.fr/prosail/). The PROSPECT model is used to simulate the leaf directional-hemispherical reflectance and transmittance as the function of four biochemical and structural parameters at the leaf level [31]. The PROSPECT model is expressed as [32]:

(2) $$\left(\rho_{1},\tau_{1}\right)=PROSPECT\left(N,C_{ab},C_{brown},C_{ar},C_{w},C_{m}\right)$$

where N is a leaf structure parameter (dimensionless), $C_{ab}$ is the chlorophyll a and *b* content (μg/cm^2^), $C_{brown}$ is the brown pigment content (μg/cm^2^), $C_{ar}$ is the carotenoid content (μg/cm^2^), $C_{w}$ is the water content (g/cm^2^), and $C_{m}$ is the dry matter content (μg/cm^2^).

The SAIL model is used to simulate the top of the canopy reflectance as a function of a series of parameters [31]. The SAIL model is expressed as [33]:

(3) $$\rho =SAIL\left(\rho_{1},\tau_{1},LAI,LAD,\rho_{soil},Diff,Hspot,SZA,VZA,RAA\right)$$

where the input parameters are: leaf reflectance $\rho_{1}$, transmittance $\tau_{1}$, leaf area index LAI, leaf inclination distribution LAD, soil coefficient $\rho_{\mathrm{soil}}$, diffuse reflection coefficient Diff, hot spot parameter Hspot, atmosphere conditions, and view-illumination geometry including solar zenith angle (SZA), view zenith angle (VZA), and relative azimuth angle (RAA).

In this study, the ranges of the $C_{ab}$, LAI, and LAD PROSAIL model parameters tested are obtained using sensitivity analysis [34,35] based on canopy reflectance simulation. The input parameters setting in the PROSAIL model are shown in Table 3.

Based on the input parameter values, running the PROSAIL model leads to the LUT for obtaining the values of the simulated LAI. Moreover, the following empirical model is developed to create the 8 m spatial resolution spatial distribution of LAI [36]:

(4) $$\hat{y}=a+\overset{n}{\underset{i=1}{\sum }}b_{i}\cdot R_{\lambda_{i}}$$

where the dependent variable ($\hat{y}$) is the estimated value of LAI, a and $b_{i}$ represent regression parameters, $R_{\lambda_{i}}$ represents the optimal relevant canopy reflectance with the greatest correlation coefficients under the condition of significant level *P* ≤ 0.05, $\lambda_{i}$ denotes spectral wavelength, and n is the total number of canopy reflectance variables. Multiple linear regression (MLR) is used to resolve the empirical estimation model to map LAI.

#### 2.4.2. Selection and Computation of Spectral Vegetation Indices

Based on previous studies about CLQ evaluation [37,38,39], the following vegetation indices including DVI, EVI, NDVI, and RVI are used to test the optimal image date in this study. The equations are expressed as [40,41,42,43]:

(5) $$DVI=R_{NIR}-R_{RED}$$

(6) $$EVI=2.5\times \frac{R_{NIR}-R_{RED}}{R_{NIR}+6R_{NIR}-7.5R_{BLUE}+1}$$

(7) $$NDVI=\frac{R_{NIR}-R_{RED}}{R_{NIR}+R_{RED}}$$

(8) $$RVI=\frac{R_{NIR}}{R_{RED}}$$

where $R_{NIR}$, $R_{RED}$ and $R_{BLUE}$ are the spectral reflectance of near-infrared, red, and blue bands, respectively, corresponding to band 4 (770–890 nm), band 3 (630–690 nm), and band 1 (450–520 nm) of GF-1 images.

In addition to the widely used vegetation indices (DVI, EVI, NDVI, and RVI), in this study, LAI is also used to test the optimal image date. The main reason for using LAI is because CLQ is a comprehensive measure of cultivated land potential productivity and LAI characterizes the canopy structure of crops that is, to a great extent, an indirect measure of CLQ. This is especially true for rice. Generally, the better the CLQ, the higher the LAI. Moreover, as the most important biophysical parameter, LAI has been widely involved in the process-based models of crop biogeochemistry and biomass.

#### 2.4.3. Determining and Validating the Optimal Image Date for Cultivated Land Quality Evaluation

For determining the optimal image date for CLQ evaluation, the most relevant image-derived spectral vegetation index is selected by analyzing the spectral response relationship between CLQ and the spectral vegetation indices from different growth stages of rice. At the present, linear, exponential, logarithmic, and power empirical models have been widely used for selecting the independent variables that have significant contributions to estimate CLQ from a large number of spectral variables [44]. The determination of the most relevant image-derived index can significantly reduce the error of model fitting and contribute the improvement of estimation accuracy. In this study, the linear, exponential, logarithmic, and power models are developed using the measured CLQ from 70 training samples as the dependent variable and each of the vegetation indices as the independent variable. This leads to 20 combinations of the vegetation indices and empirical models at each of five growth stages. As standard statistical metrics to measure model performance, RMSE and ratio of performance to deviation (RPD) are used to select the most relevant image-derived vegetation index [45].

(9) $$RMSE=\sqrt{\frac{\sum_{i=1}^{n}{\left(y_{i}-\hat{y_{i}}\right)}^{2}}{n}}$$

(10) $$RPD=\frac{STD}{RMSE}$$

where $y_{i}$ is the measured value of the ith sample; $\hat{y_{i}}$ is the estimated value of the ith sample; n is the number of samples, STD is the standard deviation of the measured CLQ. A greater RPD value indicates a higher accuracy for the quality of prediction models. The RPD > 2 corresponds to the models that can accurately predict the tested property; RPD between 1.4 and 2 indicates the models with a possible improvement, and RPD < 1.4 implies poor prediction ability of the models [46]. Then, the growth stage of rice corresponding to the most relevant vegetation index is determined as the optimal image date for CLQ evaluation.

In order to validate the reliability of the optimal image date, the model with the most accurate predictions is used to generate the spatial distribution of CLQ. The measured CLQ values of 45 validation samples are compared with the estimates from the empirical models to discriminate the accuracy of selecting the optimal image date using coefficient of determination (R^2^), mean relative error (MRE), RMSE, and relative RMSE (RRMSE) [47].

(11) $$R^{2}=1-\frac{\sum_{i=1}^{n}{\left(y_{i}-\hat{y_{i}}\right)}^{2}}{\sum_{i=1}^{n}{\left(y_{i}-\bar{y_{i}}\right)}^{2}}$$

(12) $$MRE=\frac{\sum_{i=1}^{n}\left|y_{i}-\hat{y_{i}}\right|/y_{i}}{n}$$

(13) $$RRMSE=\frac{RMSE}{\bar{y}}\times 100\%$$

where $\bar{y}$ is the mean value of the measured values.

## 3. Results

### 3.1. Estimation of Leaf Area Index

**Figure 2:**
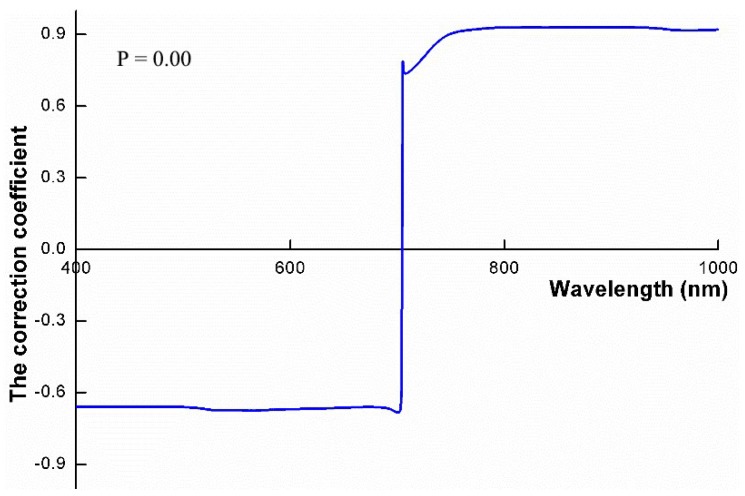
Pearson correlation coefficients between simulated leaf area index (LAI) and spectral canopy reflectance.

Based on the input parameters in Table 3, spectral canopy reflectance is simulated using the PROSAIL model. The correlation analysis is then performed on the simulated LAI and spectral canopy reflectance. The Pearson correlation coefficients are shown in Figure 2, which indicates that band 750–900 nm has the larger correlation coefficient with LAI compared to other spectral bands. Thus, GF-1 band 4 with a range of 770–890 nm is selected to map the LAI at an 8 m spatial resolution.

**Figure 3:**
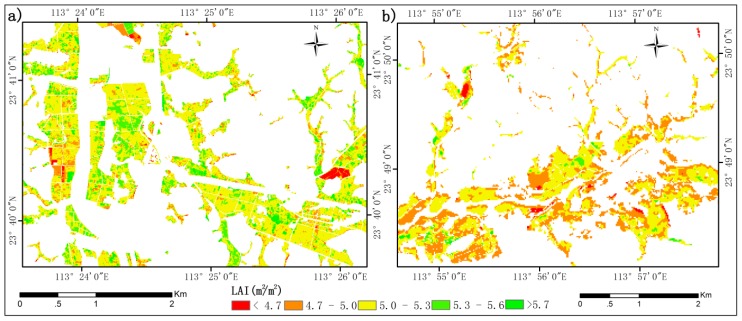
The spatial distribution of LAI retrieved using the PROSAIL model on 15 October 2015: (**a**) block 1 and (**b**) block 2.

The canopy reflectance data simulated by the PROSAIL model are spectrally resampled by the spectral resampling model in ENVI 5.3 to correspond to the spectral resolution of GF-1 band 4 (770–890 nm). Then, the resampled canopy reflectance and the simulated LAI are applied to solving Equation (4). Finally, the spatial distribution of LAI (Figure 3) is obtained.

**Figure 4:**
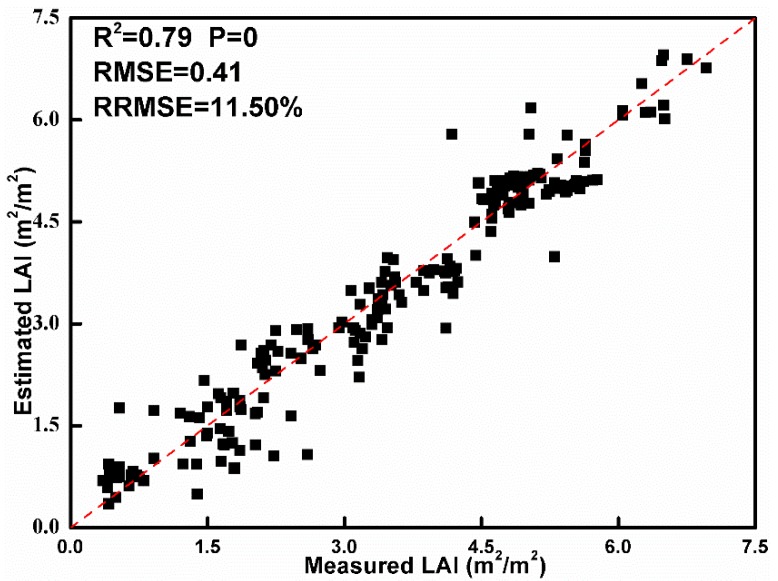
Ground-measured LAI values versus estimated LAI values using the PROSAIL model from 3 August 2015 to 24 October 2015.

The estimated values and field measured values of LAI from a total of 225 validation samples (45 samples from each of five growth stages) of rice are compared by calculating the RMSE and relative RMSE percentage (Figure 4). The results suggest that the measured and estimated LAI values are significantly correlated at the significant level of 0.05. The values of R^2^, RMSE, and RRMSE are 0.79, 0.41 m^2^/m^2^ and 11.50%, respectively, which suggests that the estimated LAI values are reliable.

### 3.2. The Optimal Image Date of Cultivated Land Quality Evaluation

**Figure 5:**
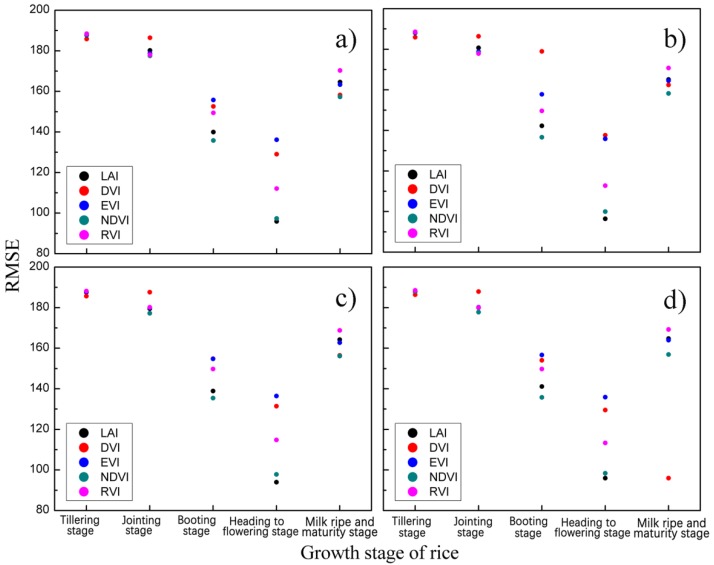
The scatterplots of root mean square error (RMSE) values of the empirical estimation models for determining the optimal image data: (**a**) linear model; (**b**) exponential model; (**c**) logarithmic model, and (**d**) power model.

**Figure 6:**
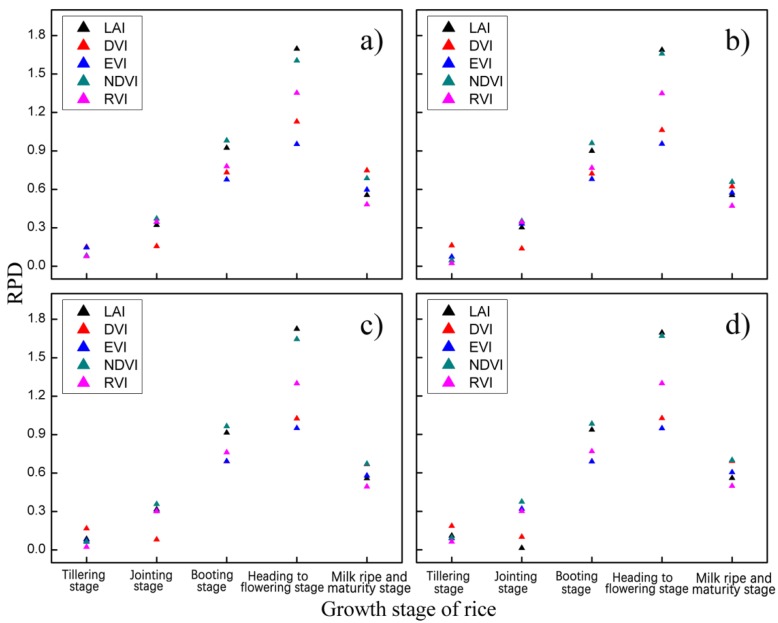
The scatterplots of ratio of performance to deviation (RPD) values from the empirical estimation models for determining the optimal image data: (**a**) linear model; (**b**) exponential model; (**c**) logarithmic model, and (**d**) power model.

To determine the optimal image date of CLQ evaluation, the RMSE and RPD values at different growth stages are calculated based on the measured and estimated CLQ values from the spectral indices-based empirical models. The similar trends of RMSE for all the vegetation indices are observed in Figure 5. All the indices show that the RMSE values decrease as the growth stage changes from tillering to jointing and booting, reaches the smallest values at the heading to flowering stage and then increases at the milk ripe and maturity stage. That is, the RMSEs of all the indices at the heading to flowering stage are smaller than those at the other growth stages, indicating that the heading to flowering stage is the optimal image date to evaluate CLQ. Moreover, the LAI-based logarithm model has the smallest RMSE compared to other indices and models, implying LAI at heading to flowering stage is the optimal spectral vegetation index to evaluate CLQ. The same result is confirmed from Figure 6 based on the maximum RPD.

**Figure 7:**
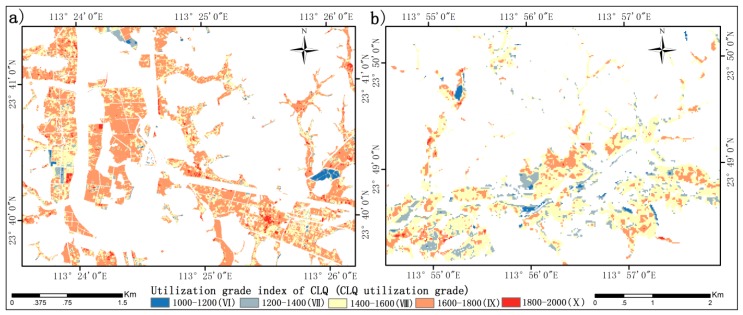
Spatial distributions of the estimated cultivated land quality (CLQ) utilization grade index: (**a**) block 1; (**b**) block 2.

**Figure 8:**
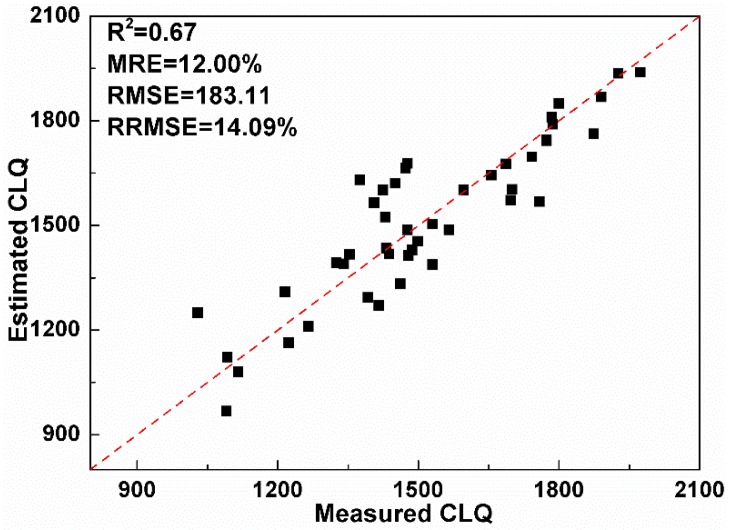
The measured versus estimated CLQ values based on the 45 validation sample plots.

In order to validate the optimal image date of CLQ evaluation, the LAI-based logarithm regression model is applied with the GF-1 image acquired at the heading to flowering stage to map CLQ in Figure 7. In order to validate the effect of the optimal image date for estimating CLQ, the accuracy metrics (R^2^, MRE, RMSE, and RRMSE) are calculated using the validation dataset. In this study, 45 soil samples (black points in Figure 1) are used to validate the estimation accuracy of CLQ based on the optimal image date. The results of comparing the estimated and measured CLQ values are shown in Figure 8, where the estimated values of CLQ are plotted against the measured values. The R^2^, MRE, RMSE, and RRMSE are respectively 0.67, 12.00%, 183.11 and 14.09%, indicating that the image date corresponding to the heading to flowering stage is optimal and could be used to evaluate CLQ.

## 4. Discussion

Previous research on determining the optimal image date [15,18,22], while providing valuable information, has some limitations. First, previous studies [8,9,10,48] mainly focus on determining the optimal image date for crop identification, crop harvest, and crop yield estimation. The results of the studies cannot be used to estimate CLQ. The reason is mainly because CLQ is a comprehensive measure of cultivated land’s potential productivity and varies depending on many factors such as climate conditions, topographic features, soil properties and environmental conditions, irrigation conditions, etc. Crop yields are related to CLQ but cannot be directly used to quantify CLQ because different crops have different amounts of yield and their yields are greatly affected by human activities, such as fertilizing and natural disasters. In addition, different crops have different spectral responses. Thus, this study is the first effort reported to determine the optimal image date for estimating CLQ and significantly contributed to the literature in this field. Based on the comparison of five vegetation indices and four estimation models, this study leads to consistent finding: the heading to flowering stage has the smallest RMSE and greatest RPD value, indicating that the heading to flowering stage is the best time to acquire images to evaluate CLQ. Among the vegetation indices and models, the LAI-based logarithmic model also provided the greatest estimation accuracy of CLQ. The results implied that the image acquired at the heading to flowering stage of rice is sensitive to CLQ and also reliable for its evaluation.

In this study, the commonly used vegetation indices DVI, EVI, NDVI, and RVI are compared with LAI and the results showed that the LAI-based logarithm model at the heading to flowering stage has the smallest RMSE. This implied that the LAI acquired at the heading to flowering stage performs better than the other vegetation indices for CLQ evaluation. This could be explained by the characteristics of LAI that quantifies canopy structure of crops. Although LAI is not a direct consequence of CLQ, generally, a high LAI value implies the good land quality. This is especially true at the heading to flowering stage of rice. On the other hand, it is well known that crops often do not grow well in the areas with poor land quality. That is, LAI can indirectly measure CLQ. Of course, the values of LAI also vary depending on plant characteristics. However, the effect of plant characteristics on LAI is relatively weak in crops especially at the heading to flowering stage of rice [49,50].

## 5. Conclusions and Future Prospect

### 5.1. Conclusions

It has been well known that evaluating and mapping CLQ using remote sensing data is a quick and effective method but very challenging due to different image dates being selected with different spectral responses, which will affect the accuracy of CLQ evaluation. For this purpose, this study focuses on determining the optimal image date of CLQ evaluation using samples taken from Conghua district of Guangzhou, Guangdong Province, China. The following conclusions could be drawn: (1) compared to other growth stages (tillering stage, jointing stage, booting stage, and milk ripe and maturity stage), the image-derived spectral indices (LAI, DVI, EVI, NDVI, and RVI) at the heading to flowering stage have smaller RMSEs and greater RPD values of estimating CLQ, indicating that the heading to flowering stage is the optimal image date for estimating and mapping high-quality CLQ; (2) among the combinations of five vegetation indices and four empirical models, the LAI-based logarithm model in the heading to flowering stage have the smallest RMSE, which imply the combination of canopy structure parameter LAI and logarithm model performed best to estimate CLQ; and (3) the validation leads to a RRMSE value of 14.09% for mapping CLQ, which further indicate that the heading to flowering stage is the optimal time period to acquire images for evaluating CLQ at the regional scale. This study is the first report that provided an effective means for determining the optimal image date of CLQ evaluation.

### 5.2. Prospect for Future Studies

It has to be pointed out that although the sampling design is conducted based on soil and land use types, the sample sizes are relatively small. The soil sample data used to determine the optimal image date have a range of CLQ from middle to high values and lack the values of low level. These might have affected the performance and accuracy of estimating CLQ. In the future studies, larger sample sizes with the values of CLQ ranging from low to high should be utilized to further determine and validate the optimal image date of CLQ evaluation. Moreover, in this study only five GF-1 images during the rice growth period are used to select the optimal image date, which might have limited the analysis of the relationships between the vegetation indices and CLQ. In the future, more images at different dates should be considered to obtain a more reliable optimal image date for CLQ evaluation.

In addition, spectral data on the basis of pixel-by-pixel of remote sensing images are used in this study and the image-derived vegetation indices are considered to be independent from each other and also to have no spatial autocorrelation. In fact, the spectral data and the image-derived vegetation induces are usually spatially auto-correlated and cross-correlated with each other [51]. Thus, the method proposed in this study might have limited the estimation accuracy of CLQ and could only be regarded as an applicable approach for determining the optimal image date for CLQ evaluation. In the future, spatial or texture based measures should be integrated with spectral vegetation indices [51] to improve the estimation of CLQ and the determination of its optimal image date.

The study serves as a reference for future work about the optimal image date of CLQ evaluation. Because of the absence of other field crop type information, we cannot currently validate the optimal image dates of CLQ evaluation and uncertainties brought about by the types of crop. Thus, more field-measured samples from different crops need to be collected to further validate the reliability of the optimal image date proposed in the study. In the next work, we will also test the capacity of LAI to estimate the different components of CLQ for determining the most relevant image date of LAI among the image dates of different CLQ components and CLQ.

## Figures and Tables

**Table 1:** Acquisition dates of GF-1 images used and corresponding with rice growth stages.

Growth Stage	Tilling Stage	Jointing to Booting Stage		Heading to Flowering Stage	Milk Ripe and Maturity Stage
		Jointing	Booting		
Acquisition date (y/m/d)	8/3/2015	9/17/2015	9/26/2015	10/15/2015	10/24/2015

**Table 2:** Radiance calibration parameter values of GF-1 satellite.

Satellite	Parameter Value	Bands			
		Band 1	Band 2	Band 3	Band 4
GF-1	Gain	0.2072	0.1776	0.177	0.1909
	Bias	7.5348	3.9395	−1.7445	−7.2053

**Table 3:** The input parameters setting in the PROSAIL model.

Model	Parameter	Symbol	Unit	Min	Max
PROSPECT	leaf structure index	N	dimensionless	1.5	1.5
	leaf chlorophyll content a + b	C_ab_	μg/cm^2^	20	80
	carotenoid content	C_ar_	μg/cm^2^	8	8
	brown pigment	C_brown_	μg/cm^2^	0	0
	water content	C_w_	g/cm^2^	0.005	0.005
	dry matter content	C_m_	μg/cm^2^	0.015	0.015
SAIL	leaf area index	LAI	m^2^/m^2^	0.05	7
	hot parameter	Hspot	m^2^/m^2^	0.2	0.2
	leaf angle distribution	LAD	°	20	50
	diffuse reflection coefficient	Diff	fraction	0.1	0.1
	soil coefficient	ρ_soil_	dimensionless	0.1	0.1
	Sun zenith angle	SZA	°	30	30
	view zenith angle	VZA	°	0	0
	relative azimuth angle	RAA	°	0	0
